# Light-Powered Self-Translation of an Asymmetric Friction Slider Using a Liquid Crystal Elastomer String Oscillator

**DOI:** 10.3390/polym16243520

**Published:** 2024-12-18

**Authors:** Dali Ge, Jiangtao Duan, Wu Bao, Haiyi Liang

**Affiliations:** 1 School of Civil Engineering, Anhui Jianzhu University, Hefei 230601, China; dalige@ahjzu.edu.cn (D.G.); duanjiangtao@stu.ahjzu.edu.cn (J.D.); wubao2001@stu.ahjzu.edu.cn (W.B.); 2 IAT-Chungu Joint Laboratory for Additive Manufacturing, Institute of Advanced Technology, University of Science and Technology of China, Hefei 241200, China; 3 CAS Key Laboratory of Mechanical Behavior and Design of Materials, Department of Modern Mechanics, University of Science and Technology of China, Hefei 230026, China

**Keywords:** asymmetric friction slider, self-translation, self-oscillation, liquid crystal elastomer, light-driven

## Abstract

In recent years, there have been many studies focused on improving the performance of active materials; however, applying these materials to active machines still presents significant challenges. In this study, we introduce a light-powered self-translation system for an asymmetric friction slider using a liquid crystal elastomer (LCE) string oscillator. The self-translation system was composed of a hollow slide, two LCE fibers, and a mass ball. Through the evolution of photothermal-induced contraction, we derived the governing equations for the system. Numerical simulations revealed two distinct motion modes: the static mode and the self-translation mode. As the mass ball moved, the LCE fibers alternated between illuminated and non-illuminated states, allowing them to effectively harvest light energy to compensate for the energy dissipation within the system. Unlike traditional self-oscillating systems that oscillate around a fixed position, the asymmetric friction enabled the slider to advance continuously through the oscillator’s symmetric self-sustained oscillation. Furthermore, we explored the critical conditions necessary for initiating self-translation as well as key system parameters that influence the frequency and amplitude of the oscillator and average speed of the slider. This self-translation system, with its simple design and ease of control, holds promising potential for applications in various fields including soft robotics, energy harvesting, and active machinery.

## 1. Introduction

Active materials have the ability to alter their shape, size, and properties in response to external stimuli such as light [1,2], electricity [3,4], magnetic fields [5], heat [6,7], and pH [8], enabling them to perform specific tasks. Typical examples of active materials include polydimethylsiloxane [9], hydrogels [10,11], liquid crystal elastomers (LCEs) [12,13,14,15,16,17], and photo-responsive or thermal responsive polymers [18,19], etc. Active materials offer numerous advantages including self-healing, self-adaptation, lightweight, and flexible structure. Active materials hold significant research potential in fields such as soft robots [20,21,22,23,24], energy harvesting devices [25,26], mechano-logistic devices [27], self-propelled devices [28,29], and more.

Given the benefits of active materials, they have extensive potential to be employed in active machines. However, to enhance their efficiency and broader application, it is essential to develop an appropriate control method. Traditional approaches for controlling active machines encompass electronic component control and programmed design control [30,31]. However, these methods exhibit limitations such as the dependence on human intervention, the need for complex control systems, and high cost. In intricate work settings, these constraints may diminish the effectiveness of active machines, raise the safety risks, and potentially disrupt their proper functioning.

To address the issues above-mentioned, we adopted a new control method akin to the functioning of human organisms: self-oscillation. Self-oscillation refers to the periodic motion of a system under a constant external stimulus [32,33,34] and it can autonomously absorb external energy to counteract damping dissipation during motion. Its amplitude and frequency are typically determined by the system’s parameters. Additionally, self-oscillation demonstrates strong robustness [35]. Thanks to these advantages, self-oscillation systems hold significant potential for a wide range of applications in fields such as autonomous robots [21,22,23,24], energy-absorbing devices [25,26], sensors [36], cargo transport [37,38] and logical operations [39,40].

In recent years, the more types of oscillation modes are available, the more sophisticated autonomous devices may potentially be constructed such as bending [41,42,43], jumping [44,45,46], rolling [47,48,49,50], swinging [51,52], stretching and contracting [53,54,55,56], twisting [57,58], vibrating [59,60] and rotation [61,62,63], and even the synchronized motion of multiple coupled self-oscillators [64]. Many of these self-sustaining motions rely on nonlinear feedback mechanisms such as self-shading [65,66], photothermal solvent evaporation [67], and photothermal surface tension gradients [68]. These mechanisms disrupt the system’s initial equilibrium, enabling the active material to respond steadily and continuously to external stimuli, resulting in self-oscillations.

Among the various types of active materials, LCEs are an integration of liquid crystal (LC) mesogens and polymer networks, characterized by rapid response speed, significant macroscopic deformation, and reversible shape transformation [4,6,12,22,47,69]. They show great potential for enabling self-oscillation in various forms [34,35,41,47,50,65], thereby providing a strong foundation for the advancement of active machines. In this paper, we introduce a light-powered self-translation of an asymmetric friction slider using an LCE string oscillator. This device incorporates two LCE fibers and an oscillator enclosed within the slider, with each LCE fiber vertically linked inside the oscillator and the slider. The slider can continuously advance while the oscillator exhibits symmetric self-sustained oscillation under constant lighting conditions, without the need for periodic excitation forces or variable stiffness [70,71,72]. In contrast to traditional active machines, this system features a simple structure, easy control, and energy efficiency. These characteristics are particularly important for applications ranging from search and rescue operations to transportation, soft robotics, and active machinery.

The rest of this article is organized as follows. In Section 2, we derive the governing equations for the self-oscillation of the oscillator and the self-translation of the slider based on the evolution of photothermal-induced contraction. Section 3 describes the two distinct modes of motion: the static mode and the self-translation mode. Meanwhile, we explore the mechanism behind the system’s self-translation motion. In Section 4, through numerical calculations, we examine how various system parameters influence the amplitude and frequency of the oscillator as well as the average velocity of the slider. Finally, Section 5 provides a summary of the findings.

## 2. Theoretical Model and Formulation

This section introduces a theoretical model for the self-translation system under steady illumination. It encompasses the dynamic governing equations for the oscillator and slider and the evolution of photothermal-induced contraction.

### 2.1. Dynamics of the Self-Translation System

**Figure 1:**
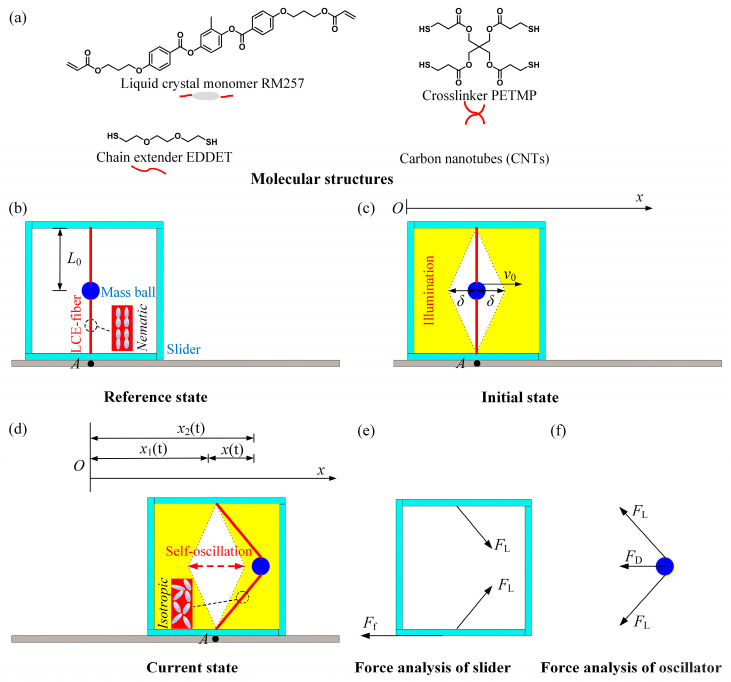
Diagram of the self-translation system including (**a**) molecular structures, (**b**) reference state, (**c**) initial state, (**d**) current state, (**e**) force analysis of slider, and (**f**) force analysis of oscillator. The slider is subject to the tension $F_{L}$ of the LCE fibers and the friction force $F_{f}$ between the slider and the contact surface, while the mass ball experiences tension $F_{L}$ from the LCE fibers and the damping force $F_{D}$. Under constant lighting conditions, the self-translation system can maintain continuous forward movement.

Figure 1 illustrates a light-powered self-translation of an asymmetric friction slider using an LCE string oscillator, which can continuously move forward, provided with a specific initial velocity of the mass ball and under predetermined lighting conditions. It comprises two photothermal-responsive LCE fibers with an original length of $L_{0}$ in stress-free state, a mass ball with a mass of m, and a slider, as depicted in Figure 1b. Each LCE fiber is anchored at one end within the slider and at the other end to the mass ball in the vertical plane. Notably, photothermal-driven LCEs have garnered significant attention. The LCE fibers in our study can be fabricated using the method described in reference [14], with the molecular structures of the components used for synthesis shown in Figure 1a. In the reference state, the LC mesogens are aligned along the length direction of the LCE fibers. Upon heating beyond the nematic-to-isotropic phase transition temperature, the LC mesogens undergo a transition from the nematic phase to the isotropic phase [2,14,47]. This transition leads to a contraction along the length direction of the LCE fibers. When the LCE fibers have cooled down, the LC mesogens will revert to their oriented alignment. As a result, LCE fibers exhibit a reversible contraction and recovery behavior triggered by temperature fluctuations.

As depicted in Figure 1c, the illumination region is represented by the yellow portion, while the other is the non-illumination region. The distance between the initial position of the mass ball and the edge of the non-illumination region is denoted as δ. In the initial state, the mass ball is given an initial velocity as $v_{0}$ to the right horizontally, causing it to move in that direction while the tension in the LCE fiber gradually increases. As the mass ball enters the illuminated region, the temperature of the LCE fibers gradually increases due to the photothermal effect. As a result, the oriented nematic mesogens become isotropic, causing the fibers to contract. This accelerates the rate of tension growth, which leads to a gradual decrease in the velocity of the mass ball until it reaches zero, after which it moves in the opposite direction. After some time, when the mass ball enters the non-illuminated region, the temperature of the LCE fibers gradually decreases, causing the LC mesogens to revert to their oriented alignment, and the photothermal-induced contraction gradually recovers. When the mass ball returns to its initial position, its inertia carries it to further move to the left. Upon entering the illumination region on the left, the LCE fibers contract again in response to light, causing the tension increase, and the velocity of the mass ball continues to decrease to a standstill. The mass ball then moves back to the initial position, even to the right.

Due to the tension from the LCE fibers, the slider may move left or right during the oscillation of the mass ball. Considering the asymmetry contacting between the slider and the contact surface, the slider experiences different frictional forces with the different friction coefficients when moving rightward and leftward. Therefore, under steady illumination, the slider may translate toward one direction accompanied with the mass ball oscillating between right and left.

To describe the system’s motion, designate point A on the contact surface as a reference, as shown in Figure 1b. During the motion, the direction of horizontal motion to the right is defined as positive. The self-translation system experiences the tension $F_{L}\left(t\right)$ of the LCE fibers and the friction force $F_{f}\left(t\right)$ between the slider and the contact surface, as illustrated in Figure 1e. It is assumed that the gravitational force mg acting on the mass ball is much less than the vertical component of the tension in the LCE fibers, and mg can be neglected. Therefore, as the mass ball moves, the symmetry of the LCE fibers connecting both ends ensures that the vertical components of tension counterbalance each other, and the mass ball moves exclusively in the horizontal direction. Thus, in the horizontal direction, the governing equations for the motion of the slider can be expressed as follows:

(1) $$\left(M-m\right){\ddot{x}}_{1}\left(t\right)=2F_{L}\left(t\right)\frac{x\left(t\right)}{\sqrt{{L_{0}}^{2}+x^{2}\left(t\right)}}-F_{f}\left(t\right),$$

where $x\left(t\right)$ indicates the relative displacement between the mass ball and the slider, M is the total weight of the self-translation system, and ${\ddot{x}}_{1}\left(t\right)$ represents the acceleration of the slider. The relationship between the tension and the elongation of the LCE fibers can be expressed as follows:

(2) $$F_{L}\left(t\right)=K\left[\sqrt{{L_{0}}^{2}+x^{2}\left(t\right)}-L_{0}-L_{0}\varepsilon \left(t\right)\right],$$

where K represents the elastic coefficient of the LCE fibers, and $\varepsilon \left(t\right)$ denotes the photothermal-induced contraction of the LCE fibers. The frictional force $F_{f}\left(t\right)$ between the slider and the surface can be described as:

(3) $$F_{f}\left(t\right)=\mu Mg\cdot \mathrm{sgn}\left({\dot{x}}_{1}\right),$$

where μ is the coefficient of friction between the system and the surface, g is the acceleration due to gravity, and $\mathrm{sgn}\left({\dot{x}}_{1}\right)$ describes the direction of the slider’s frictional force. When the slider is moved to the right, $\mathrm{sgn}\left({\dot{x}}_{1}\right)=1$ and $\mu =\mu_{1}$. When the slider is moved to the left, $\mathrm{sgn}\left({\dot{x}}_{1}\right)=-1$ and $\mu =\mu_{2}$. When the slider is stationary, $\mathrm{sgn}\left({\dot{x}}_{1}\right)=0$.

Meanwhile, the mass ball experiences tension $F_{L}\left(t\right)$ from the LCE fibers and damping force $F_{D}\left(t\right)$ experienced by the mass ball during its movement, as depicted in Figure 1f. In the horizontal direction, the governing equation for the mass ball can be expressed as:

(4) $$m{\ddot{x}}_{2}\left(t\right)=-2F_{L}\left(t\right)\frac{x\left(t\right)}{\sqrt{{L_{0}}^{2}+x^{2}\left(t\right)}}-F_{D}\left(t\right),$$

where ${\ddot{x}}_{2}\left(t\right)$ denotes the absolute acceleration of the mass ball. Based on the geometric relationship, it becomes evident that:

(5) $$x_{2}\left(t\right)=x\left(t\right)+x_{1}\left(t\right),$$

where $x_{2}\left(t\right)$ denotes the absolute displacement of the mass ball, and $x_{1}\left(t\right)$ denotes the displacement of the slider. For simplicity, the damping force of the mass ball is assumed to be proportional to its velocity, given by the formula:

(6) $$F_{D}\left(t\right)=\beta {\dot{x}}_{2}\left(t\right),$$

where β denotes the damping coefficient, and ${\dot{x}}_{2}(t)$ represents the absolute velocity of the mass ball.

To calculate the tension in Equation (2), it is necessary to first evaluate the photo-thermal-induced contraction *ε*(*t*), with the specifics outlined in the following section.

### 2.2. Photothermal-Induced Contraction

This section focuses on the photothermal-induced contraction in LCE fibers. The LCE fibers exhibited a reversible contraction and restoration response induced by changes in temperature. For simplicity, the photothermal-induced contraction $\varepsilon \left(t\right)\;$ was presumed to have a linear relationship with the temperature difference $T\left(t\right)$ of the LCE fiber [2,6,14,47], which can be expressed as follows:

(7) $$\varepsilon \left(t\right)=-CT\left(t\right),$$

where C  is the thermal contraction coefficient.

Due to the photothermal effect, the temperature of the LCE fiber increases under illuminated conditions. Considering that the length of the LCE fiber is much larger than its cross-sectional radius, we assumed that heat exchange occurs rapidly enough for the temperature within the photothermal-responsive LCE fiber to remain uniform. It is worth noting that while the LCE fiber is being heated, it simultaneously dissipates heat to the surrounding environment. Therefore, under steady illumination, the temperature difference of the LCE fiber can be expressed as

(8) $$\frac{dT\left(t\right)}{dt}=\frac{I_{0}-k_{c}T\left(t\right)}{\rho_{C}},$$

where $I_{0}$ represents the photothermal flux from the steady illumination, $k_{c}$ denotes the heat transfer coefficient, and $\rho_{C}$ is the heat capacity. Here, $T_{max}=I_{0}/k_{c}$ defines the limited temperature difference for the LCE fiber when a constant light is applied, and $\tau =\rho_{C}/k_{c}$ represents the thermal relaxation time, reflecting the rate of heat exchange between the LCE fiber and its surroundings. It is worth mentioning that in this work, the LCE fiber switched between the illuminated and non-illuminated regions. For the case of LCE fibers in the non-illuminated region, the current photothermal flux was set as $I_{0}=0$ for $x\left(t\right)<\delta$.

## 3. Two Motion Modes and Mechanism of Self-Translation

In this section, we examine the two motion modes within the LCE string oscillator and slider: the static mode and the self-translation mode. Additionally, we delve into the corresponding self-translation mechanism.

### 3.1. Two Motion Modes

According to the theoretical model for the self-translation system under steady illumination, we further nondimensionalized the governing Equations (1)–(8), as presented in Appendix A. From Appendix A, the light-powered self-translation can be determined for a given set of dimensionless parameters: ${\bar{I}}_{0}$, $\bar{C}$, $\bar{\delta }$, $\bar{K}$, ${\bar{v}}_{0}$, $\mu_{1}$, $\bar{\beta }$, R, $\bar{g}$, and λ. Thus, it is essential to determine the specific values of the dimensionless parameters required in the model. Utilizing data from existing experiments [14,73,74,75,76], we gathered the typical material properties and geometric parameters in Table 1 and the corresponding dimensionless in Table 2. Subsequently, these parameter values were used to study the light-powered self-translation system.

**Figure 2:**
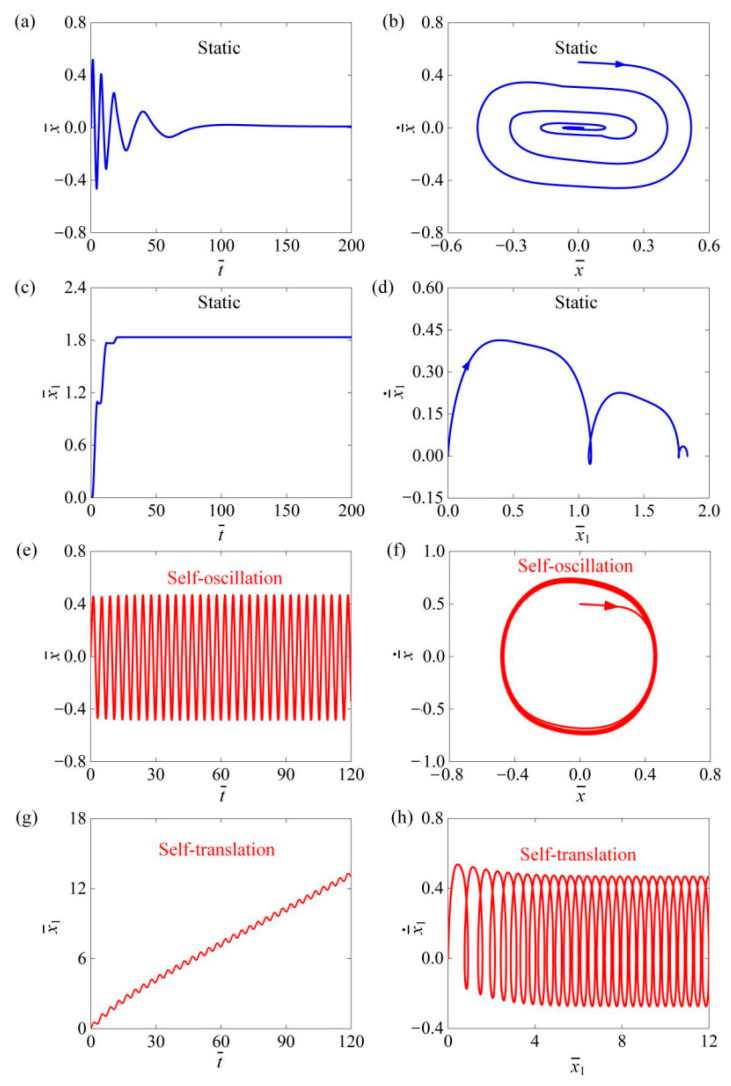
The time history curves and phase trajectories of the two motion modes of the mass ball and the slider are plotted. (**a**–**d**) The static mode with ${\bar{I}}_{0}=0$. (**e**–**h**) The self-translation mode with ${\bar{I}}_{0}=0.6$. We configured the remaining system parameters as follows: C=0.3, $\bar{K}=3.8$, $\bar{\beta }=0.08$, $\bar{\delta }=0.25$, λ=2.0, $\mu_{1}=0.01$, R=1.8, $\bar{g}=2.0$, and ${\bar{v}}_{0}=0.5$. The mass ball and slider could exhibit two types of motion modes: static mode and self-translation mode.

Figure 2 illustrates the time history curves and phase trajectories for the two motion modes of the self-translation system. The parameters used in the calculation were set as follows: C=0.3, ${\bar{I}}_{0}=0.6$, $\bar{K}=3.8$, $\bar{\beta }=0.08$, $\bar{\delta }=0.25$, λ=2.0, $\mu_{1}=0.01$, R=1.8, $\bar{g}=2.0$, and ${\bar{v}}_{0}=0.5$. The results indicate that the self-translation system exhibits two motion modes, namely the static mode and the self-translation mode. Figure 2a–d depicts the static mode of the system without photothermal flux at ${\bar{I}}_{0}=0$, where the relative displacement and velocity between the mass ball and the slider gradually diminished due to damping, and the slider moved slowly over time as a result of friction. Eventually, the oscillator and slider rested on the contact surface, which is named as the static mode. Figure 2e–h is shown as ${\bar{I}}_{0}=0.6$, where the mass ball is initially given a velocity to the right to reach the illumination region, and both the relative velocity of the mass ball and the velocity of the slider gradually increase over time, and finally remains constant. Therefore, the mass ball enters the self-oscillation mode. While the slider moves forward accompanied with oscillating back and forth, it means that the slider has achieved the motion of self-oscillation-driven translation, which is named as the self-translation mode.

### 3.2. Mechanism of Self-Translation

**Figure 3:**
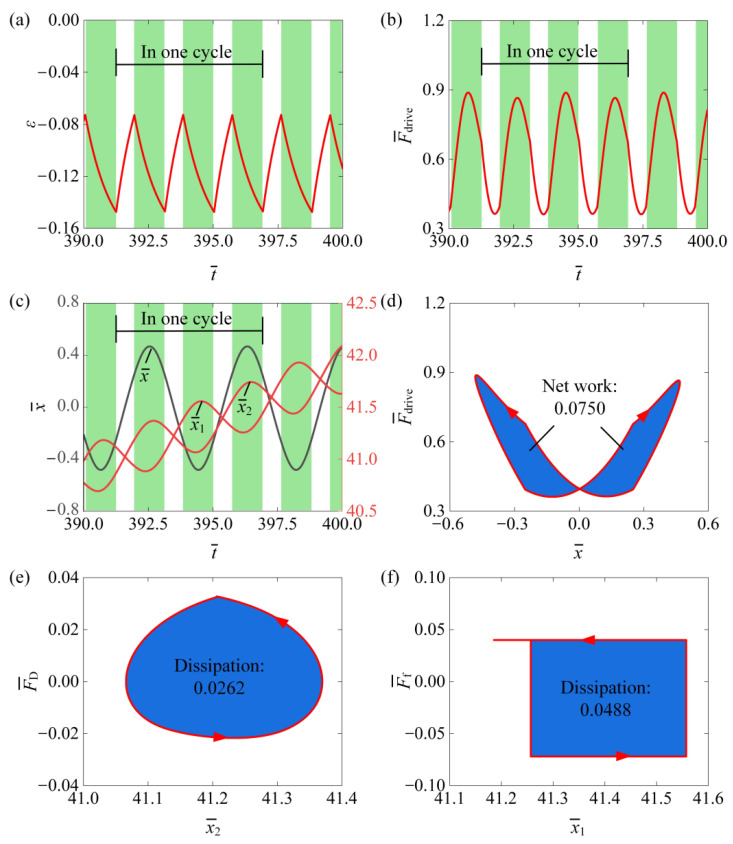
The mechanism of self-translation of an asymmetric friction slider for the typical case in Figure 2e–h. (**a**) The time history curve of ε. (**b**) The time history curve of ${\bar{F}}_{\mathrm{drive}}$. (**c**) The time history curve of $\bar{x}$, ${\bar{x}}_{1}$, ${\bar{x}}_{2}$. (**d**) The dependence between ${\bar{F}}_{\mathrm{drive}}$ and $\bar{x}$. (**e**) The dependence between ${\bar{F}}_{D}$ and ${\bar{x}}_{2}$. (**f**) The dependence between ${\bar{F}}_{f}$ and ${\bar{x}}_{1}$. The net work undertaken by ${\bar{F}}_{\mathrm{drive}}$ counteracts the energy dissipation caused by ${\bar{F}}_{D}$ and ${\bar{F}}_{f}$, thereby maintaining the self-translation.

In the light-powered self-translation of an asymmetric friction slider using an LCE string oscillator, both damping and frictional dissipation occur. We investigated the conversion between energy input energy dissipation, which can be calculated according to Appendix B. Figure 3 depicts several key physical quantities related to the self-translational motion of the slider, as observed in the typical case shown in Figure 2e,f. Figure 3a shows the photothermal-induced contraction of the LCE fibers over time, demonstrating periodic variation. The green-shaded area indicates that the LCE fibers are in the illumination region during the mass ball’s self-oscillation. Correspondingly, Figure 3b illustrates that the equivalent driving force ${\bar{F}}_{\mathrm{drive}}$ also has periodic changes over time. Figure 3c illustrates the relative displacement $\bar{x}$ between the mass ball and the slider, the displacement ${\bar{x}}_{1}$ of the slider, and the absolute displacement ${\bar{x}}_{2}$ of the mass ball over time. It is evident that the relative displacement $\bar{x}$ between the mass ball and the slider varied periodically with time, while both the displacement ${\bar{x}}_{1}$ of the slider and the absolute displacement ${\bar{x}}_{2}$ of the mass ball increased continuously over time. The dependence between the force ${\bar{F}}_{\mathrm{drive}}$ and the relative displacement $\bar{x}$ formed a clockwise closed loop during one period of the mass ball’s self-oscillating motion, as shown in Figure 3d. This closed loop represents the net work undertaken by the horizontal component of the LCE fiber tension over a cycle. The calculated value was 0.0750. Figure 3e,f illustrates the dependence between the damping force ${\bar{F}}_{D}$ and the absolute displacement ${\bar{x}}_{2}$ and the dependence between the friction ${\bar{F}}_{f}$ and the absolute displacement ${\bar{x}}_{1}$ over a self-oscillation cycle. It is evident that both the damping force and the frictional force traced out a counterclockwise closed loop over a cycle, with the areas of these loops calculated to be 0.0262 and 0.0488, respectively. Clearly, over one self-oscillation cycle, the net work undertaken by the tension of the horizontal component of the LCE fibers could entirely offset the work conducted by the damping force and the frictional force. This balance ensures the self-translation of the entire system under constant photothermal flux conditions.

## 4. Impact of System Parameters on the Self-Translation

The following dimensionless parameters exist in the above theoretical model including ${\bar{I}}_{0}$, $\bar{C}$, $\bar{K}$, $\bar{\beta }$, $\bar{\delta }$, λ, $\mu_{1}$, R, $\bar{g}$, and ${\bar{v}}_{0}$. In this section, we examine the trigger conditions for self-translation as well as the dimensionless frequency f and amplitude A of the mass ball’s self-oscillation and the dimensionless average speed ${\bar{v}}_{a}$ of the slider’s self-translation.

### 4.1. Influence of Photothermal Flux

**Figure 4:**
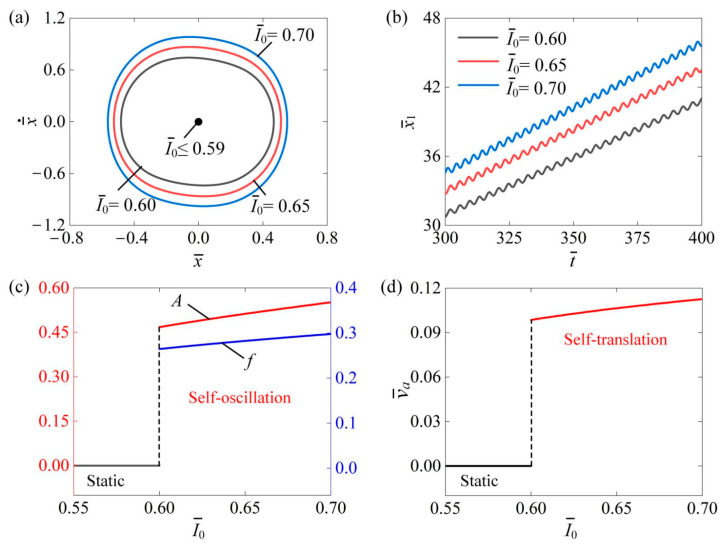
The effect of photothermal flux ${\bar{I}}_{0}$ on the self-translation system, with the remaining system parameters set as follows: $\bar{C}=0.3$, $\bar{K}=3.8$, $\bar{\beta }=0.08$, $\bar{\delta }=0.25$, λ=2.0, $\mu_{1}=0.01$, R=1.8, $\bar{g}=2.0,$ and ${\bar{v}}_{0}=0.5$. (**a**) The limiting circles of the mass ball. (**b**) Displacement–time curves of the slider. (**c**) The amplitude and frequency of self-oscillation. (**d**) The average velocity of self-translation. As photothermal flux increased, an upward trend was observed in both the A and f of the mass ball’s self-oscillation as well as in the ${\bar{v}}_{a}$ of the slider’s self-translation.

Figure 4 shows the impact of photothermal flux ${\bar{I}}_{0}$ on the self-translation system. The remaining system parameters are as follows: $\bar{C}=0.3$, $\bar{K}=3.8$, $\bar{\beta }=0.08$, $\bar{\delta }=0.25$, λ=2.0, $\mu_{1}=0.01$, R=1.8, $\bar{g}=2.0$, and ${\bar{v}}_{0}=0.5$. Figure 4a displays the limiting circles of the mass ball’s self-oscillation at three different light intensities: ${\bar{I}}_{0}=0.6$, ${\bar{I}}_{0}=0.65$, and ${\bar{I}}_{0}=0.7$. A critical photothermal flux of approximately 0.59 was identified for triggering self-translation. When the photothermal flux is above this threshold, the energy input to the system compensates for the energy dissipation, enabling self-translation. Notably, the size of the limit circle increases with rising photothermal flux during self-translation. Figure 4b illustrates the displacement ${\bar{x}}_{1}$ of the slider over time at ${\bar{I}}_{0}=0.6$, ${\bar{I}}_{0}=0.65$, and ${\bar{I}}_{0}=0.7$. As the photothermal flux increases, the slider’s displacement also rises over the same duration. In Figure 4c, it is evident that both the amplitude A and frequency f increased with the increase in ${\bar{I}}_{0}$. This enhancement occurs because higher photothermal flux amplifies the photothermal-induced contraction of the LCE fibers, thereby generating a greater equivalent driving force on the slider, which enables it to perform more net work. Furthermore, as shown in Figure 4d, the average speed ${\bar{v}}_{a}$ of the slider correspondingly increased with the photothermal flux. These results suggest that increasing the photothermal flux can enhance the engineering applications of the slider’s self-translation.

### 4.2. Influence of Contraction Coefficient

**Figure 5:**
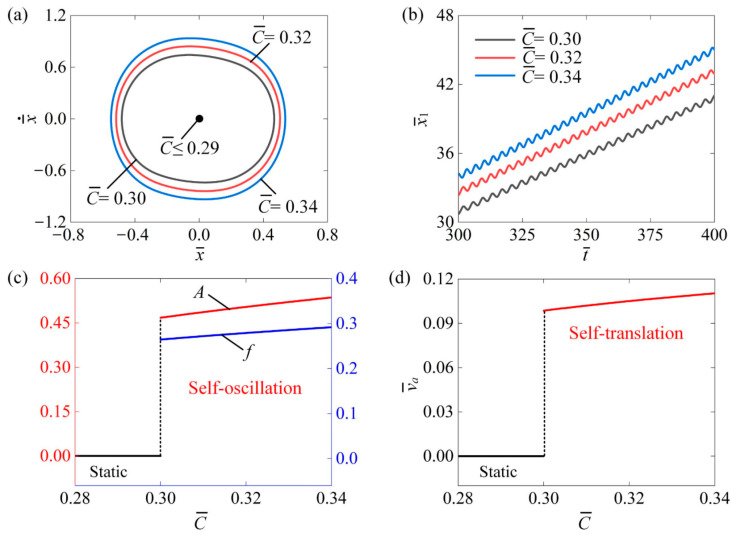
The impact of the contraction coefficient of the LCE fibers on the self-translation system. The remaining system parameters are configured as follows: ${\bar{I}}_{0}=0.6$, $\bar{K}=3.8$, $\bar{\beta }=0.08$, $\bar{\delta }=0.25$, λ=2.0, $\mu_{1}=0.01$, R=1.8, $\bar{g}=2.0,$ and ${\bar{v}}_{0}=0.5$ (**a**) The limiting circle of the mass ball. (**b**) Displacement–time curves of the slider. (**c**) The amplitude and frequency of self-oscillation. (**d**) The average velocity of self-translation. As the contraction coefficient of the LCE fibers increased, both the A and f of the mass ball’s self-oscillation as well as the ${\bar{v}}_{a}$ of the slider’s self-translation, demonstrated an upward trend.

Figure 5 explores the effect of the contraction coefficient C of the LCE fiber on the self-translation system. The other system parameters are as follows: ${\bar{I}}_{0}=0.6$, $\bar{K}=3.8$, $\bar{\beta }=0.08$, $\bar{\delta }=0.25$, λ=2.0, $\mu_{1}=0.01$, R=1.8, $\bar{g}=2.0$, and ${\bar{v}}_{0}=0.5$. Figure 5a shows the limiting circles of the mass ball’s self-oscillation at three different contraction coefficients, namely $\bar{C}=0.3$, $\bar{C}=0.32$, and $\bar{C}=0.34$. A critical contraction coefficient of approximately 0.29 was identified as the threshold for triggering self-translation. When the contraction coefficient exceeds this threshold, the energy input to the system offsets energy dissipation, facilitating self-translation. Importantly, the size of the limit circle increases with a higher contraction coefficient during self-translation. Figure 5b depicts the displacement ${\bar{x}}_{1}$ of the slider over time at $\bar{C}=0.3$, $\bar{C}=0.32$, and $\bar{C}=0.34$. As the contraction coefficient rises, the displacement ${\bar{x}}_{1}$ of the slider also increases over the same period. In Figure 5c, it is clear that both the amplitude A and frequency f increased with the contraction coefficient. This enhancement occurs because greater contraction coefficient amplifies the photothermal-induced contraction of the LCE fibers, resulting in a stronger equivalent driving force on the slider, which allows it to perform more net work. Additionally, as illustrated in Figure 5d, the average speed ${\bar{v}}_{a}$ of the slider correspondingly rose with an increasing contraction coefficient. These findings indicate that enhancing the contraction coefficient can improve the efficient conversion of energy from light to mechanical energy.

### 4.3. Influence of Non-Illuminated Width

**Figure 6:**
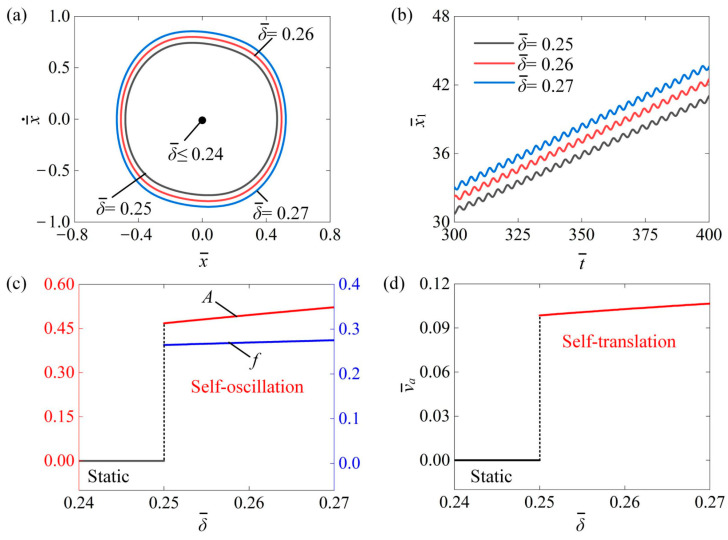
The influence of non-illuminated width on the self-translation system. The remaining system parameters are configured as follows: ${\bar{I}}_{0}=0.6$, $\bar{C}=0.3$, $\bar{K}=3.8$, $\bar{\beta }=0.08$, λ=2.0, $\mu_{1}=0.01$, R=1.8, $\bar{g}=2.0,$ and ${\bar{v}}_{0}=0.5$. (**a**) The limiting circle of the mass ball. (**b**) Displacement–time curves of the slider. (**c**) The amplitude and frequency of self-oscillation. (**d**) The average velocity of self-translation. Increasing the non-illuminated width resulted in an increase in self-oscillation A, f, and translational ${\bar{v}}_{a}$.

Figure 6 discusses how the non-illuminated width $\bar{\delta }$ affects the self-translation system. In the calculation, we set ${\bar{I}}_{0}=0.6$, $\bar{C}=0.3$, $\bar{K}=3.8$, $\bar{\beta }=0.08$, λ=2.0, $\mu_{1}=0.01$, R=1.8, $\bar{g}=2.0$, and ${\bar{v}}_{0}=0.5$. Figure 6a depicts the size of the limit cycles under three different non-illuminated widths for $\bar{\delta }=0.25$, $\bar{\delta }=0.26,$ and $\bar{\delta }=0.27$. A critical non-illuminated width of about 0.24 can be obtained to trigger self-translation. When $\bar{\delta }<0.24$, the system is in a static mode, while the system is in the self-translation mode when $\bar{\delta }>0.24$. This is because a larger non-illuminated width provides more time for the LCE fibers to recover from the photothermal-induced contraction, allowing them to absorb more light energy in the illuminated region. Figure 6b presents the time history curve of displacement ${\bar{x}}_{1}$ of the slider under the non-illuminated width of $\bar{\delta }=0.25$, $\bar{\delta }=0.26,$ and $\bar{\delta }=0.27$. It can be easily observed that the displacement ${\bar{x}}_{1}$ of the slider increased as $\bar{\delta }$ increased. Figure 6c shows that as $\bar{\delta }$ increased, the self-oscillation amplitude A and frequency f increased. Figure 6d shows that with the increase in the non-illuminated width, the average speed of the slider also increased. For the self-translation system, it is essential to induce photothermal-induced contraction in the illuminated region. Additionally, sufficient time for recovery from this contraction is also required. As the photothermal-induced contraction approaches its maximum value (illustrated in Figure 3a), increasing the non-illuminated width can enhance the recovery process. This, in turn, allows for a greater absorption of light energy in the illuminated region. Therefore, increasing the non-illuminated width results in an increase in self-oscillation amplitude A, frequency f, and translational average speed ${\bar{v}}_{a}$.

### 4.4. Influence of Elastic Coefficient

**Figure 7:**
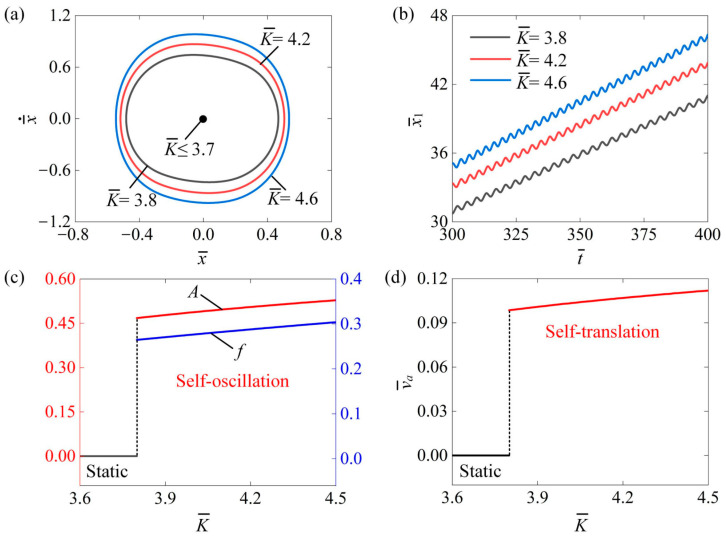
The impact of the elastic coefficient of the LCE fibers on self-translation. The remaining system parameters are defined as follows: ${\bar{I}}_{0}=0.6$, $\bar{C}=0.3$, $\bar{\delta }=0.25$, $\bar{\beta }=0.08$, λ=2.0, $\mu_{1}=0.01$, R=1.8, $\bar{g}=2.0,$ and ${\bar{v}}_{0}=0.5$. (**a**) The limiting circle of the mass ball. (**b**) Displacement–time curves of the slider. (**c**) The amplitude and frequency of self-oscillation. (**d**) The average velocity of self-translation. As the elasticity coefficient increases, the system demonstrated a noticeable rise in the A and f of the mass ball’s self-oscillation motion and the ${\bar{v}}_{a}$ of the slider’s self-translation motion.

Figure 7 examines the impact of the coefficient of elasticity $\bar{K}$ on the self-translation system under parameters such as$\;{\bar{I}}_{0}=0.6$, $\bar{C}=0.3$, $\bar{\delta }=0.25$, $\bar{\beta }=0.08$, λ=2.0, $\mu_{1}=0.01$, R=1.8, $\bar{g}=2.0,$ and ${\bar{v}}_{0}=0.5$. Figure 7a illustrates the limit circles of the mass ball’s self-oscillation at three different elasticity coefficients $\bar{K}=3.8$, $\bar{K}=4.2$, and $\bar{K}=4.6$. The results indicate that there is a critical value of 3.8 that transitions the mass ball from a static state to a self-oscillation state. When the $\bar{K}$ is below this critical value, the mass ball remains static; however, when it exceeds the critical value, the mass ball enters a state of self-oscillation. As the $\bar{K}$ increases, the boundary of the self-oscillation of the mass ball also expands. Figure 7b displays the absolute displacement ${\bar{x}}_{1}$ of the slider’s self-translation over time for different elasticity coefficients as $\;\bar{K}=3.8$, $\bar{K}=4.2$, and $\bar{K}=4.6.\;$Clearly, as the $\bar{K}$ increased, the ${\bar{x}}_{1}$ also rose. This can be attributed to the fact that a higher $\bar{K}$ enhances the equivalent driving force of the LCE fibers, enabling the slider to cover greater distances. Figure 7c demonstrates the effect of $\bar{K}$ on the amplitude A and frequency f of the mass ball’s self-oscillation. Figure 7d shows how $\bar{K}$ influences the average speed ${\bar{v}}_{a}$ of the slider’s self-translation. As $\bar{K}$ increases, A and f, along with ${\bar{v}}_{a}$, all rise. This suggests that a higher $\bar{K}$ results in a greater equivalent driving force from the LCE fibers, leading to increased A, f, and ${\bar{v}}_{a}$.

### 4.5. Influence of Initial Velocity

**Figure 8:**
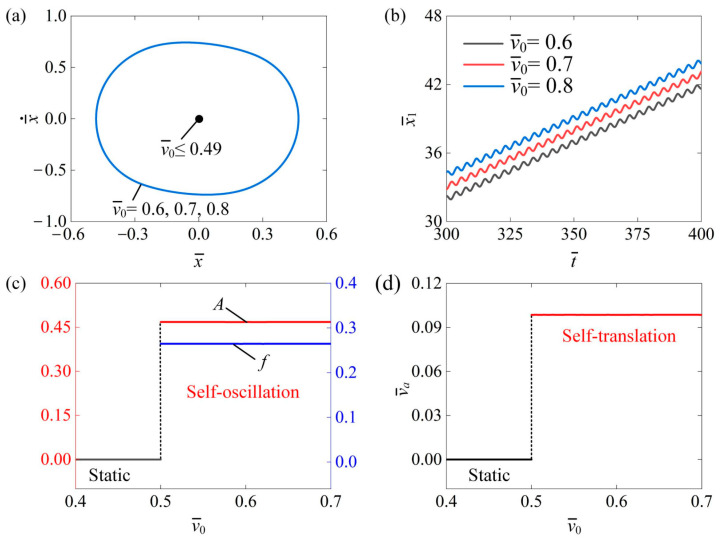
The impact of initial velocity on the self-translation system. The other parameters of the system are configured as follows: ${\bar{I}}_{0}=0.6$, $\bar{C}=0.3$, $\bar{\delta }=0.25$, $\bar{K}=3.8$, $\bar{\beta }=0.08$, λ=2.0, $\mu_{1}=0.01$, $\bar{g}=2.0,$ and R=1.8. (**a**) The limiting circle of the mass ball. (**b**) Displacement-time curves of the slider. (**c**) The amplitude and frequency of self-oscillation. (**d**) The average velocity of self-translation. As the initial velocity increases, the A and f of the mass ball’s self-oscillation and the ${\bar{v}}_{a}$ of the slider’s self-translation tend to be constant.

Figure 8 shows the influence of the initial velocity ${\bar{v}}_{0}$ on the self-translation system. In the calculation, we set ${\bar{I}}_{0}=0.6$, $\bar{C}=0.3$, $\bar{\delta }=0.25$, $\bar{K}=3.8$, $\bar{\beta }=0.08$, λ=2.0, $\mu_{1}=0.01$, $\bar{g}=2.0,$ and R=1.8. Figure 8a plots the limit circles of the mass ball’s self-oscillation relevant to different initial velocities. A critical velocity ${\bar{v}}_{0}$ of about 0.49 existed in the phase transition between static mode and self-translation mode while ${\bar{v}}_{0}<0.49$ exhibited static mode due to the fact that insufficient energy was input to compensate for the damping dissipation. The self-translation mode was triggered at ${\bar{v}}_{0}=0.6$, ${\bar{v}}_{0}=0.7,$ and ${\bar{v}}_{0}=0.8$, which had an identical limit circle, as shown in Figure 8a. Figure 8b depicts the absolute displacement ${\bar{x}}_{1}$ of the slider’s self-translation over time. It was observed that during the same time period, an increase in the initial velocity corresponded to an increase in the slider’s displacement. This indicates that a higher initial velocity results in greater initial kinetic energy within the system, allowing the slider to cover a longer distance during its translation motion. Figure 8c illustrates how the amplitude A and frequency f change with ${\bar{v}}_{0}$. Both the A and f of self-oscillation displayed no variation along with ${\bar{v}}_{0}$. Figure 8d illustrates how the average velocity ${\bar{v}}_{a}$ of the slider’s self-translation varied with the initial velocity ${\bar{v}}_{0}$. Once the system is in the self-translation state, the average velocity ${\bar{v}}_{a}$ will also remain unaffected. This is due to the fact that the initial conditions do not influence the behavior of self-oscillation, as these are intrinsic characteristics of the process. Consequently, the self-translation driven by continuous oscillation is likewise unaffected by the initial conditions.

### 4.6. Influence of the Coefficient of the Force of Friction

**Figure 9:**
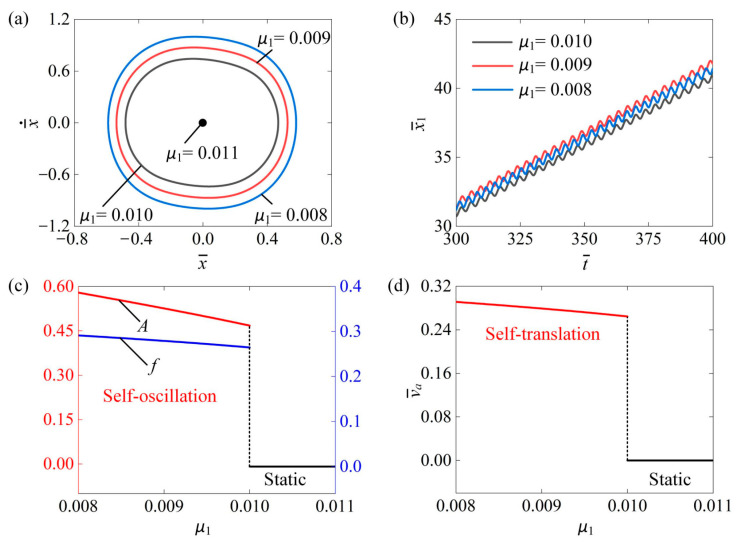
The impact of friction coefficient $\mu_{1}$ on the self-translation system under the given parameters of ${\bar{I}}_{0}=0.6$, $\bar{C}=0.3$, $\bar{\delta }=0.25$, $\bar{\beta }=0.08$, λ=2.0, $\bar{K}=3.8$, R=1.8, $\bar{g}=2.0,$ and ${\bar{v}}_{0}=0.5$. (**a**) The limiting circle of the mass ball. (**b**) Displacement–time curves of the slider. (**c**) The amplitude and frequency of self-oscillation. (**d**) The average velocity of self-translation. As the friction coefficient increased, both the A, the f and the ${\bar{v}}_{a}$ of slider exhibited a downward trend.

In this section, we explore the impact of friction coefficient $\mu_{1}$ on the self-translation system. In the calculation, we set ${\bar{I}}_{0}=0.6$, $\bar{C}=0.3$, $\bar{\delta }=0.25$, $\bar{\beta }=0.08$, λ=2.0, $\bar{K}=3.8$, R=1.8, $\bar{g}=2.0,$ and ${\bar{v}}_{0}=0.5$. Figure 9a depicts the limit circles of the mass ball’s self-oscillation for three different friction coefficients: $\mu_{1}=0.01$, $\mu_{1}=0.009$, and $\mu_{1}=0.008$. A critical friction coefficient can be numerically calculated for triggering self-translation, with a value about 0.012. This outcome indicates that the slider remains in a static pattern when $\mu_{1}\geq 0.011$ and transitions to a self-translation pattern when $\mu_{1}\leq 0.011$. This is because as the friction coefficient increases, the net work generated by the equivalent driving force is no longer sufficient to counteract the damping dissipation necessary for maintaining self-translation. Figure 9b presents the time history curve of the slider’s displacement under the three friction coefficients of 0.01, 0.009, and 0.008. As the friction coefficient decreases, the displacement of the slider within the same time increases. Figure 9c illustrates that as the friction coefficient increased, both the self-oscillation amplitude and frequency decreased. This phenomenon can be attributed to the fact that as the friction coefficient increases, more energy is dissipated in the system due to friction, resulting in a smaller amplitude. Furthermore, the average speed of the slider also decreases with the increase in friction coefficient, as shown in Figure 9d. Therefore, it can be concluded that a smaller friction coefficient allows the slider to reach a self-translation pattern more easily.

### 4.7. Influence of Damping Coefficient

**Figure 10:**
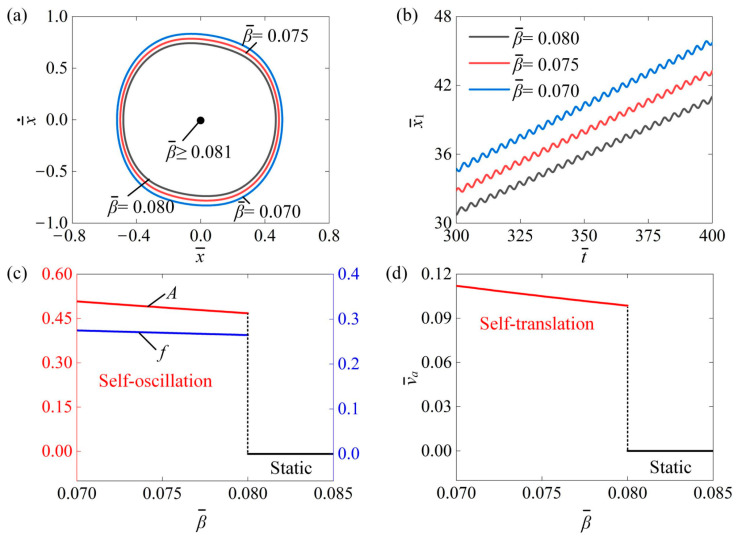
The impact of damping coefficient $\bar{\beta }$ on the self-translation system under the given parameters of ${\bar{I}}_{0}=0.6$, $\bar{C}=0.3$, $\bar{\delta }=0.25$, $\mu_{1}=0.01$, λ=2.0, $\bar{K}=3.8$, R=1.8, $\bar{g}=2.0,$ and ${\bar{v}}_{0}=0.5.$ (**a**) The limiting circle of the mass ball. (**b**) Displacement–time curves of the slider. (**c**) The amplitude and frequency of self-oscillation. (**d**) The average velocity of self-translation. As the damping coefficient increased, both the A and f of the mass ball and the ${\bar{v}}_{a}$ of slider showed a decreasing trend.

Figure 10 analyzes the impact of the damping coefficient $\bar{\beta }$ on the self-translation of the slider. The parameters were set for ${\bar{I}}_{0}=0.6$, $\bar{C}=0.3$, $\bar{\delta }=0.25$, $\mu_{1}=0.01$, λ=2.0, $\bar{K}=3.8$, R=1.8, $\bar{g}=2.0,$ and ${\bar{v}}_{0}=0.5$. Figure 10a describes the three limit cycles of the slider at the damping coefficients of $\bar{\beta }=0.08$, $\bar{\beta }=0.075$, and $\;\bar{\beta }=0.07$. Observations indicated that the size of the limit cycle increased as the damping coefficient decreased, and there was a critical damping coefficient of about 0.081 for triggering self-translation. This is because the energy input to the system was insufficient to counteract the damping dissipation for $\bar{\beta }\geq 0.081$, resulting in the system being in a static pattern. The input of energy to the system was capable of compensating for the dissipation caused by damping for $\bar{\beta }\leq 0.08$, thus the system was in a self-translation pattern. In Figure 10b, the time history curve of the slider’s displacement is shown under the damping coefficients of 0.08, 0.075, and 0.07. These findings suggest that as the damping coefficient decreases, the slider travels greater distances simultaneously. Figure 10c shows that as the damping coefficient increased, the self-oscillation amplitude A decreased, while the frequency f nearly remained constant. This occurs because a larger damping coefficient leads to greater energy dissipation within the system, which in turn produces a smaller amplitude. Additionally, it can be noted that the self-oscillation frequency remains nearly constant, regardless of changes in the damping coefficient. This is because the damping coefficient does not affect the inherent period. Moreover, as the damping coefficient increases, the average speed of the slider decreases, as illustrated in Figure 10d. To enable quicker self-translation in engineering applications, we can consider reducing the damping coefficient appropriately.

### 4.8. Influence of Friction Coefficient Ratio

**Figure 11:**
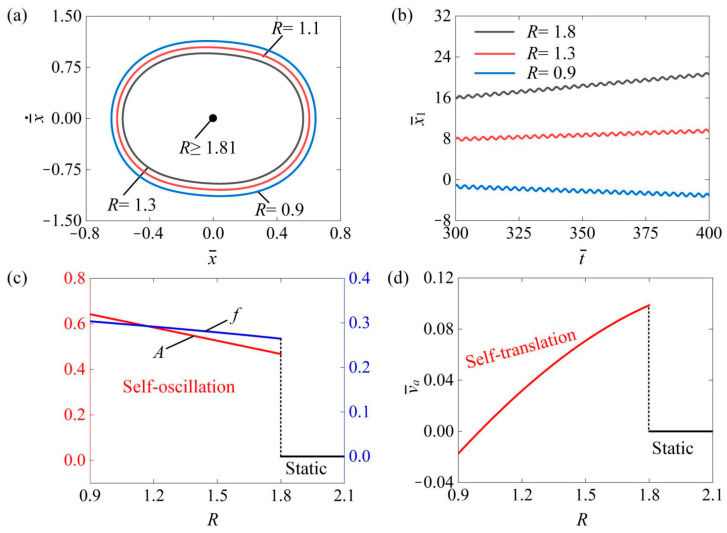
The impact of the friction coefficient ratio R on the self-translation system. The remaining system parameters are configured as follows: ${\bar{I}}_{0}=0.6$, $\bar{C}=0.3$, $\bar{\delta }=0.25$, $\mu_{1}=0.01$, λ=2.0, $\bar{K}=3.8$, $\bar{\beta }=0.08$, $\bar{g}=2.0,$ and ${\bar{v}}_{0}=0.5$. (**a**) The limiting circle of the mass ball. (**b**) Displacement–time curves of the slider. (**c**) The amplitude and frequency of self-oscillation. (**d**) The average velocity of self-translation. As the R increased, both the A and f of the mass ball’s self-oscillation exhibited a declining trend. At R<1, the absolute value of the slider’s ${\bar{v}}_{a}$ in self-translation decreases as the R increases. In contrast, at R>1, the absolute value of the average velocity ${\bar{v}}_{a}$ increases with a higher R.

Figure 11 investigates the influence of the friction coefficient ratio R on the self-translation system. The parameters were as follows: ${\bar{I}}_{0}=0.6$, $\bar{C}=0.3$, $\bar{\delta }=0.25$, $\mu_{1}=0.01$, λ=2.0, $\bar{K}=3.8$, $\bar{\beta }=0.08$, $\bar{g}=2.0,$ and ${\bar{v}}_{0}=0.5$. Figure 11a illustrates the limit circles of the mass ball’s self-oscillation under three different friction coefficients ratios of R=1.3, R=1.1, and R=0.9. It is clear that the limiting circle of the mass ball’s self-oscillation decreases as the R increases. There was a critical value of 1.81 that triggered the transition of the mass ball from a self-oscillation state to a static state. When the R was below 1.81, the net work carried out by the equivalent driving force ${\bar{F}}_{\mathrm{drive}}$ was sufficient to compensate for damping dissipation, allowing the mass ball to maintain a self-oscillation state. Conversely, when the R exceeded 1.81, the damping dissipation surpassed the net work undertaken by the ${\bar{F}}_{\mathrm{drive}}$, resulting in the mass ball becoming static. In Figure 11b, we observed the absolute displacement ${\bar{x}}_{1}$ of the slider’s self-translation over time during the same period in the  R=1.3, R=1.1, and R=0.9. Over the same time period, an increase in R resulted in a decrease in ${\bar{x}}_{1}$. This can be attributed as follows: as the R increases, the rising friction force, which increases the work and damping dissipation, ultimately hinders the slider’s movement and reduces its travel distance. Notably, when R exceeds 1, the slider moves forward; otherwise, it exhibits backward motion during self-translation. Figure 11c illustrates how the amplitude A and frequency f of the mass ball’s self-oscillation changed with the R. When the R was below the critical value of 1.81, the mass ball remained in a state of self-oscillation. In contrast, when R exceeded this critical value, the mass ball entered a static state. Figure 11d displays the average velocity of the slider’s self-translation in relation to R. When the R exceeded the critical value of 1.81, the slider remained in a static state. Conversely, if the R was below 1.81, the slider remained in a self-translation state. As the R increased, the A and f of the mass ball decreased. This can be understood as the increasing friction leading to greater work undertaken against damping dissipation, resulting in lower A and f of the mass ball’s self-oscillation. When R<1, the absolute value of the slider’s average speed decreases as the R increases. Conversely, when R>1, the absolute value of the slider’s average speed increases as the R increases. This occurs because in the case of R<1, during the slider’s self-translation, the resistance to the right is greater than that to the left, causing the slider to move left. As the R increases, the resistance to leftward movement also increases, resulting in a smaller average speed of the slider’s self-translation. In contrast, at R>1, the resistance to the left is greater than that to the right, leading the slider to move right. Here, a higher R also increases the resistance to leftward movement, which in turn results in a higher average speed of the slider’s self-translation.

### 4.9. Influence of the Mass Ratio

**Figure 12:**
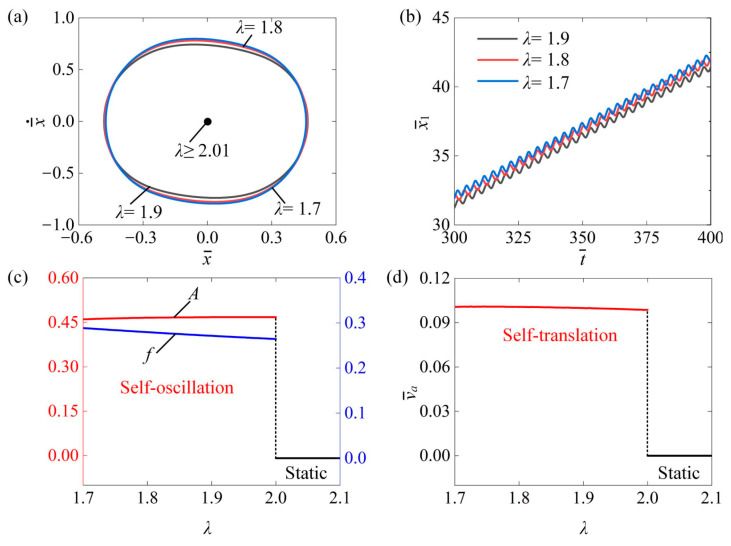
The impact of mass ratio λ on the self-translation system. The remaining system parameters are configured as follows: ${\bar{I}}_{0}=0.6$, $\bar{C}=0.3$, $\bar{\delta }=0.25$, $\mu_{1}=0.01$, R=1.8, $\bar{K}=3.8$, $\bar{\beta }=0.08$, $\bar{g}=2.0,$ and ${\bar{v}}_{0}=0.5$. (**a**) The limiting circle of the mass ball. (**b**) Displacement–time curves of the slider. (**c**) The amplitude and frequency of self-oscillation. (**d**) The average velocity of self-translation. As the mass ratio increased, it was noticeable that the amplitude A remained relatively constant, while the frequency f and the ${\bar{v}}_{a}$ of the slider’s self-translation decreased.

This section investigates the impact of mass ratio λ on the self-translation system for the given parameters of ${\bar{I}}_{0}=0.6$, $\bar{C}=0.3$, $\bar{\delta }=0.25$, $\mu_{1}=0.01$, R=1.8, $\bar{K}=3.8$, $\bar{\beta }=0.08$, $\bar{g}=2.0,$ and ${\bar{v}}_{0}=0.5$. Figure 12a plots the limit cycle under different mass ratios of λ=1.9, λ=1.8, and λ=1.7. It can be clearly seen that the size of the limit cycle decreased as the mass ratio increased, and there was a critical mass ratio of about 2.01 for triggering self-translation. When $\bar{\lambda }\geq 2.01$, the input energy to the system was insufficient to counteract the dissipation caused by damping, leading to a static state. Conversely, when $\bar{\lambda }\leq 2.01$, the input energy could adequately offset the damping dissipation, allowing the system to enter a self-translation pattern. Figure 12b presents the displacement–time curves of the slider of 1.9, 1.8, and 1.7. As the mass ratio decreased, the displacement of the slider became farther at the same time. Additionally, as shown in Figure 12c, the amplitude A remained relatively constant as the mass ratio increased, while the frequency f decreased with a rising mass ratio. This happens because, as the mass ratio of the slider to the ball increases, the friction force between the slider’s body and the contact surface also rises. Consequently, the system dissipates more energy due to this friction, resulting in a decreased frequency of the ball. In addition, the average speed of the slider also decreases, as shown in Figure 12d. Therefore, decreasing the mass ratio can improve the self-translation of the slider.

### 4.10. Influence of the Gravitational Acceleration

**Figure 13:**
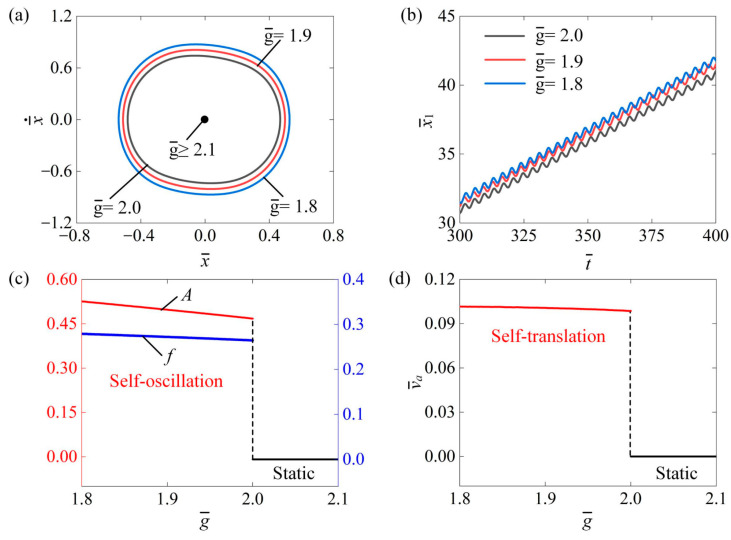
The impact of gravitational acceleration on the self-translation system. The remaining system parameters are configured as follows: ${\bar{I}}_{0}=0.6$, $\bar{C}=0.3$, $\bar{\delta }=0.25$, $\mu_{1}=0.01$, R=1.8, $\bar{K}=3.8$, $\bar{\beta }=0.08$, λ=2.0, and ${\bar{v}}_{0}=0.5$. (**a**) The limiting circles of the mass ball. (**b**) Displacement–time curves of the slider. (**c**) The amplitude and frequency of self-oscillation. (**d**) The average velocity of self-translation. As gravitational acceleration increased, there was a decreasing trend observed in both the A and f of the mass ball’s self-oscillation as well as in the ${\bar{v}}_{a}$ of the slider’s self-translation.

Figure 13 investigates the impact of gravitational acceleration $\bar{g}$ on the self-translation system. The remaining system parameters were as follows: ${\bar{I}}_{0}=0.6$, $\bar{C}=0.3$, $\bar{\delta }=0.25$, $\mu_{1}=0.01$, R=1.8, $\bar{K}=3.8$, $\bar{\beta }=0.08$, λ=2.0, and ${\bar{v}}_{0}=0.5$. Figure 13a displays the limiting circles of the mass ball’s self-oscillation at three different gravitational accelerations: $\bar{g}=1.8$, $\bar{g}=1.9$, and $\bar{g}=2.0$. A critical gravitational acceleration of approximately 2.01 was identified for triggering self-translation. When the gravitational acceleration was below this threshold, the energy input to the system offset the energy dissipation, allowing for self-translation to occur. Notably, as gravitational acceleration decreased during self-translation, the size of the limit circle also expanded. Figure 13b illustrates the displacement ${\bar{x}}_{1}$ of the slider over time at $\bar{g}=1.8$, $\bar{g}=1.9$, and $\bar{g}=2.0$. As the gravitational accelerations decreased, the slider’s displacement rose over the same duration. In Figure 13c, it is evident that both the amplitude A and frequency f decreased with the increase in ${\bar{I}}_{0}$. This occurs because heightened gravitational acceleration leads to an increase in total weight, drag, work, and damping dissipation, which collectively reduce the amplitude and frequency of the mass ball’s self-oscillating motion and the slider’s average velocity. Furthermore, as shown in Figure 13d, the average speed of the slider correspondingly decreased with gravitational accelerations. These results suggest that decreasing gravitational accelerations can enhance the engineering applications of the slider’s self-transl ation.

In summary, this section provides a systematic analysis of how key dimensionless system parameters influence the amplitude A and frequency f of the mass ball and the average velocity ${\bar{v}}_{a}$ of the slider, with the results summarized in Table 3.

## 5. Conclusions

Active materials can convert various forms of energy into mechanical energy. However, effectively utilizing these materials to implement and control active machines presents significant challenges. In this work, we leveraged a self-sustained LCE string oscillator to introduce a light-powered self-translation system for an asymmetric friction slider. The self-translation system consisted of a hollow slide, two LCE fibers, and a mass ball. Through the evolution of photothermal-induced contraction, we developed the governing equations for the self-translation system. Under constant illumination, numerical computations revealed the emergence of two distinct motion modes: static mode and self-translation mode. The motion of the mass ball causes the LCE fibers to switch between illuminated and non-illuminated states, leading to alternating photothermal-induced contraction and recovery. This allows the LCE fibers to harvest light energy, which counterbalances the energy dissipation of the self-translation system. Consequently, the self-translation of the slider can be sustained in conjunction with the self-oscillation of the oscillator.

In addition, the critical conditions necessary for triggering self-translation were identified through numerical calculation. Furthermore, we investigated how key system parameters influence the amplitude and frequency of the mass ball’s self-oscillation as well as the average speed of the slider’s self-translation. Unlike most self-oscillating systems that oscillate around a fixed position, this self-translation system utilizes an asymmetric friction structure, enabling the slider to move continuously forward through the oscillator’s self-sustained oscillation. In future work, we may further investigate the self-translation using a liquid crystal elastomer string oscillator through experimental validation. The design of this self-translation system is straightforward and easy to control, offering promising application potential across various domains including soft robotics, energy harvesting, and active machinery.

## Figures and Tables

**Table 1:** Material properties and geometric parameters.

Parameter	Definition	Value	Units
$I_{0}$	Photothermal flux	0~500	W/m^2^
C	Contraction coefficient	0~5 × 10^−3^	/°C
K	Elastic coefficient	1~3	N/m
τ	Thermal drive relaxation time	0.01~0.1	s
m	Mass of the mass ball	0~10	g
M	Mass of the self-translation system	0~10	g
β	Damping coefficient	0~0.5	mg·mm/s
$v_{0}$	Initial velocity	0~0.1	m/s
δ	Non-illuminated width	0~0.1	m
$L_{0}$	Original length of LCE fiber	0~0.2	m
$\mu_{1}$	Coefficient of forward friction	0~0.2	
$\mu_{2}$	Coefficient of receding friction	0~0.2	

**Table 2:** Dimensionless parameters.

Parameter	${\bar{I}}_{0}$	$\bar{K}$	$\bar{\beta }$	$\bar{\delta }$	λ	R	${\bar{v}}_{0}$	$\bar{g}$
Value	0~1	0~10	0~0.1	0~0.5	1~3	0.1~2	0~1	1~3

**Table 3:** Effects of several key dimensionless parameters.

Dimensionless Parameter	Amplitude A	Frequency f	$\mathrm{Average}\;\mathrm{Velocity}\;{\bar{v}}_{a}$
${\bar{I}}_{0}$	Increases with increasing ${\bar{I}}_{0}$	Increases with increasing ${\bar{I}}_{0}$	Increases with increasing ${\bar{I}}_{0}$
$\bar{C}$	Increases with increasing $\bar{C}$	Increases with increasing $\bar{C}$	Increases with increasing $\bar{C}$
$\bar{\delta }$	Increases with increasing $\bar{\delta }$	Increases with increasing $\bar{\delta }$	Increases with increasing $\bar{\delta }$
$\bar{K}$	Increases with increasing $\bar{K}$	Increases with increasing $\bar{K}$	Increases with increasing $\bar{K}$
${\bar{v}}_{0}$	Constant as ${\bar{v}}_{0}$ increases	Constant as ${\bar{v}}_{0}$ increases	Constant as ${\bar{v}}_{0}$ increases
$\mu_{1}$	Decreases with increasing $\mu_{1}$	Decreases with increasing $\mu_{1}$	Decreases with increasing $\mu_{1}$
$\bar{\beta }$	Decreases with increasing $\bar{\beta }$	Decreases with increasing $\bar{\beta }$	Decreases with increasing $\bar{\beta }$
R	Decreases with increasing R	Decreases with increasing R	Decreases with increasing R
λ	Constant as λ increases	Decreases with increasing λ	Decreases with increasing λ
$\bar{g}$	Decreases with increasing $\bar{g}$	Decreases with increasing $\bar{g}$	Decreases with increasing $\bar{g}$

## Data Availability

The original contributions presented in the study are included in the article; further inquiries can be directed to the corresponding author.

## Appendix A. Nondimensionalization

To simplify the calculation, we introduced the following dimensionless parameters: $\bar{x}=x/L_{0}$, $\dot{\bar{x}}=\dot{x}\tau /L_{0}$, $\ddot{\bar{x}}=\ddot{x}{T_{0}}^{2}/L_{0}$, ${\bar{x}}_{1}=x_{1}/L_{0}$, ${\dot{\bar{x}}}_{1}={\dot{x}}_{1}\tau /L_{0}$, ${\ddot{\bar{x}}}_{1}={\ddot{x}}_{1}\tau^{2}/L_{0}$, ${\bar{x}}_{2}=x_{2}/L_{0}$, ${\dot{\bar{x}}}_{2}={\dot{x}}_{2}\tau /L_{0}$, ${\ddot{\bar{x}}}_{2}={\ddot{x}}_{2}\tau^{2}/L_{0}$, $\bar{\delta }=\delta /L_{0}$, $\bar{t}=t/\tau$, ${\bar{F}}_{L}=F_{L}\tau^{2}/mL_{0}$, ${\bar{F}}_{f}=F_{f}\tau^{2}/mL_{0}$, ${\bar{F}}_{D}=F_{D}\tau^{2}/mL_{0}$, ${\bar{F}}_{\mathrm{drive}}=F_{\mathrm{drive}}\tau^{2}/mL_{0}$, $\bar{K}=K\tau^{2}/m$, $\bar{g}=g\tau^{2}/L_{0}$, $\bar{\beta }=\beta \tau /m$, λ=M/m, $R=\mu_{2}/\mu_{1}$, ${\bar{I}}_{0}=I_{0}/k_{c}T_{0}$, $\bar{T}=T/T_{0}$, and $\bar{C}={CT}_{0}$ ($T_{0}$ is ambient temperature). Hence, the governing Equations (1) and (4) can be expressed in dimensionless forms as follows:

(A1) $$2{\bar{F}}_{L}\left(\bar{t}\right)\frac{\bar{x}\left(\bar{t}\right)}{\sqrt{1+{\bar{x}}^{2}\left(\bar{t}\right)}}-{\bar{F}}_{f}\left(\bar{t}\right)-\left(\lambda -1\right){\ddot{\bar{x}}}_{1}\left(\bar{t}\right)=0,$$

(A2) $${\ddot{\bar{x}}}_{2}\left(\bar{t}\right)=-2{\bar{F}}_{L}\left(\bar{t}\right)\frac{\bar{x}\left(\bar{t}\right)}{\sqrt{1+{\bar{x}}^{2}\left(\bar{t}\right)}}-{\bar{F}}_{D}\left(\bar{t}\right)=0,$$

where ${\bar{F}}_{L}\left(\bar{t}\right)$, ${\bar{F}}_{f}\left(\bar{t}\right),$ and ${\bar{F}}_{D}\left(\bar{t}\right)$ can be represented by the following equations:

(A3) $${\bar{F}}_{L}\left(\bar{t}\right)=\bar{K}\left[\sqrt{1+{\bar{x}}^{2}\left(\bar{t}\right)}-1-\varepsilon \left(\bar{t}\right)\right],$$

(A4) $${\bar{F}}_{f}\left(\bar{t}\right)=\mu \lambda \bar{g}\cdot \mathrm{sgn}\left({\dot{x}}_{1}\right),$$

(A5) $${\bar{F}}_{D}\left(\bar{t}\right)=\bar{\beta }{\dot{\bar{x}}}_{2},$$

where the light-induced contraction is

(A6) $$\varepsilon \left(\bar{t}\right)=-\bar{C}\bar{T}\left(\bar{t}\right).$$

The dimensionless temperature difference in Equation (A6) can be determined as

(A7) $$\frac{d\bar{T}\left(\bar{t}\right)}{d\bar{t}}={\bar{I}}_{0}-\bar{T}\left(\bar{t}\right).\;$$

For the case of $\bar{x}\left(t\right)<\bar{\delta }$, the photothermal flux in Equation (A7) was set as ${\bar{I}}_{0}=0$. It is worth noting that the relative displacement $\bar{x}\left(\bar{t}\right)$ between the mass ball and the slider is:

(A8) $$\bar{x}\left(\bar{t}\right)={\bar{x}}_{2}\left(\bar{t}\right)-{\bar{x}}_{1}\left(\bar{t}\right),$$

Equations (A1), (A2), and (A7) govern the light-powered self-translation of an asymmetric friction slider using an LCE string oscillator, where the temperature difference of the LCE fiber is coupled with the relative displacement between the mass ball and the slider. Solving these complex differential equations with variable coefficients using analytical methods is challenging. Therefore, we opted to utilize the *Runge–Kutta* method for numerical computation, implemented through *MATLAB2017b* software. We set the initial conditions for numerical computation. For the temperature difference ${\bar{T}}_{i-1}$, the relative displacement ${\bar{x}}_{i-1}$, and the slider’s speed ${\dot{\bar{x}}}_{2\left(i-1\right)}$ at time ${\bar{t}}_{i-1}$, the tension ${{\bar{F}}_{L}}_{i-1}$ of the LCE fiber and the friction force ${{\bar{F}}_{f}}_{i-1}$ of the slider can be determined by solving Equations (A1)–(A4). Then, the current absolute displacement ${\bar{x}}_{2i}$, the displacement ${\bar{x}}_{1i}$, and the speed ${\dot{\bar{x}}}_{2i}$ at time ${\bar{t}}_{i}$ can be determined from Equations (A1) and (A2). Subsequently, the current relative displacement ${\bar{x}}_{i}$ can be calculated from Equation (A8), and the current temperature difference $\bar{T}$ can be obtained from Equation (A7). Then, the tension ${{\bar{F}}_{L}}_{i}$ and the friction force ${{\bar{F}}_{f}}_{i}$ can be obtained from Equations (A1)–(A4) again. By conducting iterative computations in *MATLAB2017b* software, we could determine the light-powered self-translation of an asymmetric friction slider using an LCE string oscillator under the specified dimensionless parameters ${\bar{I}}_{0}$, $\bar{C}$, $\bar{\delta }$, $\bar{K}$, ${\bar{v}}_{0}$, $\mu_{1}$, $\bar{\beta }$, R, $\bar{g}$, and λ.

## Appendix B. Energy Input and Energy Output

The system’s energy input $W_{\mathrm{in}}$ can be derived from the net work performed by the tension of LCE fibers. Based on the force analysis of the mass ball and slider in the dynamic model, the energy input $W_{\mathrm{in}}$ can be calculated as:

(A9) $$W_{\mathrm{in}}=\int F_{\mathrm{drive}}\left(t\right)dx_{2}-\int F_{\mathrm{drive}}\left(t\right)dx_{1},$$

where the equivalent driving force $F_{\mathrm{drive}}\left(t\right)$ is given as:

(A10) $$F_{\mathrm{drive}}\left(t\right)=2K\left[\sqrt{{L_{0}}^{2}+x^{2}\left(t\right)}-L_{0}-L_{0}\varepsilon \left(t\right)\right]\frac{x\left(t\right)}{\sqrt{{L_{0}}^{2}+x^{2}\left(t\right)}}.$$

Therefore, Equation (A9) can be rewritten as:

(A11) $$W_{\mathrm{in}}=\int F_{\mathrm{drive}}\left(t\right)dx.$$

Additionally, the energy output $W_{\mathrm{out}}$ is the sum of the system’s damping and frictional dissipation, and can be calculated as:

(A12) $$W_{\mathrm{out}}=\int F_{D}\left(t\right)dx_{2}+\int F_{f}\left(t\right)dx_{1}.$$

Defining the dimensionless energy input ${\bar{W}}_{\mathrm{in}}=\frac{W_{\mathrm{in}}}{m\tau^{2}/{L_{0}}^{2}}$ and energy output ${\bar{W}}_{\mathrm{out}}=\frac{W_{\mathrm{out}}}{m\tau^{2}/{L_{0}}^{2}}$, Equations (A11) and (A12) can be rewritten as:

(A13) $${\bar{W}}_{\mathrm{in}}=\int {\bar{F}}_{\mathrm{drive}}\left(\bar{t}\right)d\bar{x}.$$

and

(A14) $${\bar{W}}_{\mathrm{out}}=\int {\bar{F}}_{D}\left(\bar{t}\right)d{\bar{x}}_{2}+\int {\bar{F}}_{f}\left(\bar{t}\right)d{\bar{x}}_{1},$$

where ${\bar{F}}_{\mathrm{drive}}\left(\bar{t}\right)$ can be represented by the following equation:

(A15) $${\bar{F}}_{\mathrm{drive}}\left(\bar{t}\right)=2\bar{K}\left[\sqrt{1+{\bar{x}}^{2}\left(\bar{t}\right)}-1-\varepsilon \left(\bar{t}\right)\right]\frac{\bar{x}\left(\bar{t}\right)}{\sqrt{1+{\bar{x}}^{2}\left(\bar{t}\right)}},$$
